# Brief report of the first cured 2019-nCoV pneumonia patient in West China Hospital

**DOI:** 10.1007/s10096-020-03866-z

**Published:** 2020-03-27

**Authors:** En-Qiang Chen, Li-Chun Wang, Guang-Min Tang, Yao Yang, Min-Jin Wang, Rong Deng, Fang Chen, Meng-Lan Wang, Ya-Chao Tao, Ping Feng, Hong Tang

**Affiliations:** 1 Center of Infectious Diseases, West China Hospital of Sichuan University, Chengdu, 610041 China; 2 Department of Laboratory Medicine, West China Hospital of Sichuan University, Chengdu, 610041 China

## Introduction

In December 2019, the Chinese city of Wuhan has reported an outbreak of atypical pneumonia caused by the 2019 novel coronavirus (2019-nCoV). Though the detection of viral nucleic acid in respiratory tract and blood specimens is recommended for the evaluation basis for the existence or elimination of virus, and several antiviral agents and traditional Chinese medicine have been suggested for therapy [1], there are still many dilemmas in the diagnosis and individualized treatment. This report describes the clinical and laboratory characteristics of the first cured patient of 2019-nCoV infection confirmed in West China Hospital of Sichuan University.

## Case presentation

A 34-year-old man with fever (37.8 °C) and asthenia for 1 day was admitted to West China Hospital on January 22, 2020, and he came to Chengdu from Wuhan 1 day ago. In the emergency department, the screening of common respiratory viruses was negative, blood routine examination reported a significant increasing of monocyte count, and chest computed tomography (CT) reported a ground glass shadow in the right upper lobe tip segment of the lung. After admission, 2019-nCoV-RNA was detected in throat swabs. After reporting to the local Centers for Disease Control and Prevention (CDC), this patient was asked to transfer to a specialized hospital designated by the government. When he transferred to the designated hospital on January 25, the local CDC recollected the throat swab samples and re-detected 2019-nCoV-RNA. Surprisingly, 2019-nCoV-RNA was not detected in two consecutive throat swabs with an interval of 1 day. Thus, this patient was released from isolation.

**Fig. 1:**
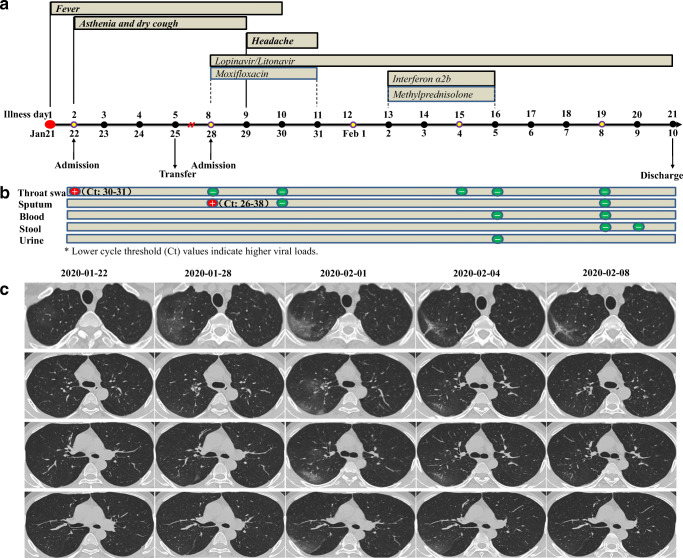
The symptoms (**a**), 2019-nCoV-RNA detection (**b**), and dynamic CT scan (**c**) according to clinical course of disease. The 2019-nCoV-RNA was quantified by real-time RT-PCR. The primers and probe targeting to ORF1ab and N gene were used. Lower cycle threshold (Ct) values indicate higher viral loads, and Ct values more than 40 indicate indeterminate

After discharge, the patient’s temperature was still abnormal, accompanied by dizziness and headache. Thus, he re-visited our hospital and was admitted again on January 28. Considering the previous detection of 2019-nCoV-RNA in throat swabs, we recollected throat swab as well as deep sputum samples. Undoubtedly, 2019-nCoV-RNA was detected in sputum at this time. On January 28, a slight aggravation of pneumonia was reported; antiviral drug lopinavir/ritonavir (two tablets twice a day) was prescribed. After 3 days’ therapy, the situation of pneumonia became worse; thus, methylprednisolone (40 mg intravenous drip for 3 consecutive days) and aerosol interferon therapies (5 million units per time for 3 consecutive days) were added. As expected, pneumonia improved significantly. On February 8, 2019-nCoV-RNA were all negative in throat swabs, sputum, blood, and feces (Fig. 1b). On February 9, he had a normal temperature without respiratory symptoms for at least 10 days, and 2019-nCoV-RNA was still negative in feces. Thus, he was discharged and asked to observe at home for 2 weeks. The detailed clinical information was shown in Fig. 1. During hospitalization, the results of noninvasive finger pulse oxygen saturation were in normal range, even when there is significant progress of pneumonia in chest CT.

## Discussion

Though clinical cure standard of 2019-nCoV pneumonia is issued by the National Health Commission, there are still many important issues to be concerned about. According to the experience on the diagnosis and treatment of this patient, we would discuss the following problems encountered in practice.

The first question is how to provide a qualified specimen for viral existence evaluation. Collecting the right specimen at the right time is extremely important. Bronchoalveolar lavage fluid (BALF) is the best specimen type, but it is not realistic for almost all hospitals. In recently published literature, saliva, nasopharynx swab, sputum, blood, and feces samples are mentioned, but only nasopharynx swabs and sputum are routinely used. The 2019-nCoV in nasopharynx swab or sputum is recommended as the judgment standard of virus infection and clearance. For this case, the initial diagnosis of 2019-nCoV infection and the followed denial of 2019-nCoV infection in other hospital are both based on the results of 2019-nCoV detection in pharyngeal swabs. What is the cause for the negative result in other hospital? In addition to the possible deficiencies in sample collection and processing, the impact of disease’s own progress also cannot be ignored. The longer the course of disease, the more likely the virus will disappear in throat swab. Thus, the detection of nasopharynx swab in early stage of diseases may be of more value than in late stage of diseases, and it is inappropriate to use nasopharynx swab result to determine whether the virus is eliminated in the body. For patients with negative viral detection in nasopharyngeal swab, but with a clear epidemiological history or typical clinical manifestations, we cannot easily deny or exclude the possibility of 2019-nCoV infection.

Compared with the negative pharyngeal swab detection on second admission of this patient, a positive detection in sputum is enough to reflect the advantage of sputum in determining virus infection. As long as conditions permit, we strongly recommend sputum for virus detection. If necessary, we can also use some special equipment or inducer to help us collect enough sputum for measurement. Of course, for patients who really cannot provide sputum samples, nasal swabs are better than pharyngeal swabs for viral nucleic acid detection.

The possibility of virus in saliva, urine, and feces is also of great concern [2, 3], especially in feces samples. At discharge, 2019-nCoV was not detected in the feces of this patient. But we cannot rule out the possibility of virus detection in his feces after discharge in the future. So, the second question is how to evaluate the results of viral nucleic acid detection in feces. Recently, scientists have isolated 2019-nCoV strains from critically ill patients’ feces. In our hospital, 2019-nCoV was not detected in blood of all admitted patients (unpublished data). Thus, 2019-nCoV in feces may come from sputum swallowed by mouth, rather than from blood. But we do not know whether the persistent positive feces detection means the long-term carrying of the virus. At present, if conditions permit, it is better to do a fecal 2019-nCoV detection before de-isolation. If the detection result is positive, we may consider extending the time for isolation in hospital. Currently, negative fecal detection is required for de-isolation in our hospital. However, we still need to remind that this proposal lacks sufficient evidence at present and its rationality needs to be further confirmed.

The mortality rate of critical patients is very high; thus, it is important to be conscious of disease progress in early stage. So, the third question is how to early predict the rapidly worsening conditions. Besides respiratory symptoms, pulmonary CT and arterial blood gas analysis are two tools for evaluating the patients’ condition, and dynamic chest CT is easier to achieve for most hospitals. In this patient, the judgment of disease progression mainly depends on the deterioration of pulmonary HRCT, even without a significant increase in respiratory rate and a decrease in blood oxygen saturation. In fact, pulmonary CT is strongly recommended for assessing the presence and severity of pneumonia [4]. Thus, at the early stage of pneumonia, pulmonary CT should be done every 3–5 days, and this could help to accurately predict the worsening of pneumonia.

Glucocorticoids have long been controversial in viral pneumonia treatment, and the pessimistic effects of glucocorticoid come from the treatment of critical pneumonia patients with large-dose and long-term using [5]. For 2019-nCoV pneumonia, glucocorticoids are not mentioned in such a patients whose condition is developing to severe or critical stage [1]. In this patient, low-dose and short-course glucocorticoids were prescribed, and a rapid pulmonary HRCT improvement was observed. Glucocorticoids can reduce pulmonary inflammatory reaction and improve symptoms of hypoxia and respiratory distress [6]. Thus, timely, appropriate, and short-term use of glucocorticoids may be helpful for avoiding excessive lung damage.

In summary, we report the first cured patient in West China Hospital, and some realistic problems have been raised during the diagnosis and treatment. We found that the detection of nasopharynx swab in early stage of diseases may be of more value than in late stage of diseases, and it is inappropriate to use nasopharynx swab result to determine whether the virus is eliminated in the body. As long as conditions permit, we strongly recommend collecting sputum for virus nucleic acid detection. At the early stage of pneumonia, the judgment of disease progression could depend on the deterioration of pulmonary HRCT, even without a significant increase in respiratory rate and a decrease in blood oxygen saturation.

## References

[1] Lin L, Li TS (2020) Interpretation of “Guidelines for the Diagnosis and Treatment of Novel Coronavirus (2019-nCoV) Infection by the National Health Commission (Trial Version 5)”. Zhonghua Yi Xue Za Zhi 100 (0):E001 10.3760/cma.j.issn.0376-2491.2020.0001 32033513

[2] To KK, Tsang OT, Chik-Yan Yip C, Chan KH, Wu TC, Chan JMC, Leung WS, Chik TS, Choi CY, Kandamby DH, Lung DC, Tam AR, Poon RW, Fung AY, Hung IF, Cheng VC, Chan JF, Yuen KY (2020) Consistent detection of 2019 novel coronavirus in saliva. Clin Infect Dis 10.1093/cid/ciaa149 PMC7108139 32047895

[3] Holshue ML, DeBolt C, Lindquist S, Lofy KH, Wiesman J, Bruce H, Spitters C, Ericson K, Wilkerson S, Tural A, Diaz G, Cohn A, Fox L, Patel A, Gerber SI, Kim L, Tong S, Lu X, Lindstrom S, Pallansch MA, Weldon WC, Biggs HM, Uyeki TM, Pillai SK, Washington State -nCo VCIT (2020) First case of 2019 novel coronavirus in the United States. N Engl J Med 10.1056/NEJMoa2001191 PMC7092802 32004427

[4] Kanne JP (2020) Chest CT findings in 2019 novel coronavirus (2019-nCoV) infections from Wuhan, China: key points for the radiologist. Radiology:200241 10.1148/radiol.2020200241 PMC7233362 32017662

[5] Oray M, Abu Samra K, Ebrahimiadib N, Meese H, Foster CS (2016). Long-term side effects of glucocorticoids. Expert Opin Drug Saf.

[6] Vandewalle J, Luypaert A, De Bosscher K, Libert C (2018). Therapeutic mechanisms of glucocorticoids. Trends Endocrinol Metab.

